# Epithelioid inflammatory myofibroblastic sarcoma: a case report and brief literature review

**DOI:** 10.3389/fonc.2023.1212529

**Published:** 2023-09-29

**Authors:** Weidong Dou, Yu Guan, Tao Liu, Hang Zheng, Shuo Feng, Yingchao Wu, Xin Wang, Zhanbing Liu

**Affiliations:** Department of General Surgery, Peking University First Hospital, Peking University, Beijing, China

**Keywords:** EIMS, ALK-inhibitors, ALK, case report, literature review

## Abstract

Epithelioid inflammatory myofibroblastic sarcoma (EIMS) is a rare variant of the inflammatory myofibroblastic tumor, characterized by more aggressive clinical course and nuclear membrane staining of anaplastic lymphoma kinase (ALK) with ALK rearrangement. An elderly male came to the clinic because of an accidental abdominal mass. Abdominal and pelvic enhanced CT revealed a tumor apparently orginated from mesenchymal tissue. Subsequently, the abdominal mass and multiple organ resection was performed, and the mass was pathologically confirmed as EIMS. The patient developed Clavien-Dindo Grade III postoperative complications and was discharged after his condition improved. He received doxorubicin monotherapy after operation, but only one cycle was administered due to severe vomiting. The follow-up of 5 months after operation showed no evidence of recurrence. Given the rarity of EIMS, and ALk inhibitors have a long and robust effect on patients with ALK gene tumors, it is very important for clinicians to be familiar with the clinicopathological features of EIMS, which will contribute to the accurate diagnosis of EIMS and reduce misdiagnosis.

## Introduction

Inflammatory myofibroblastic tumor (IMT) is a distinctive mesenchymal-derived tumor characterized by predominantly myofibroblastic spindle cells with inflammatory infiltration, which may recur and rare metastasize (1). ALK rearrangement on chromosome 2p23 is found in 50% of IMT patients (2, 3). Epithelioid inflammatory myofibroblastic sarcoma (EIMS), known as a variant of IMT, which was first described by Marino-Enriquez in 2011 (4), exhibits malignant behavior, has a high likelihood of recurrence and is associated with a poor prognosis (5, 6). Most of the literatures focus on the pathological features of EIMS. Herein, we report a case of EIMS and review the previous literature to summarize its clinical features and treatment progress.

## Case presentation

**Figure 1:**
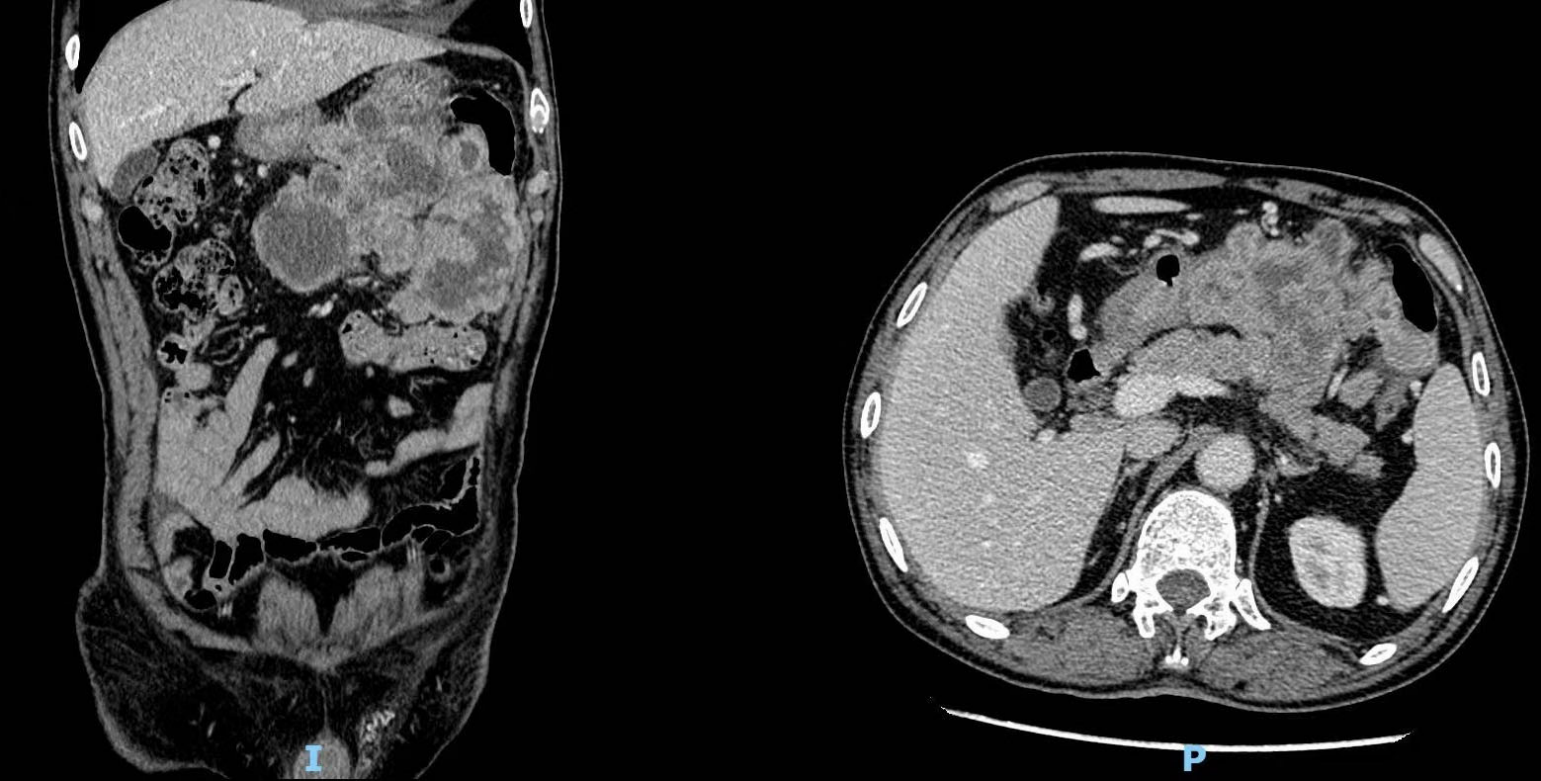
Computed tomography (CT) revealed multiple soft-tissue density masses in the left upper abdomen with unclear boundaries.

**Figure 2:**
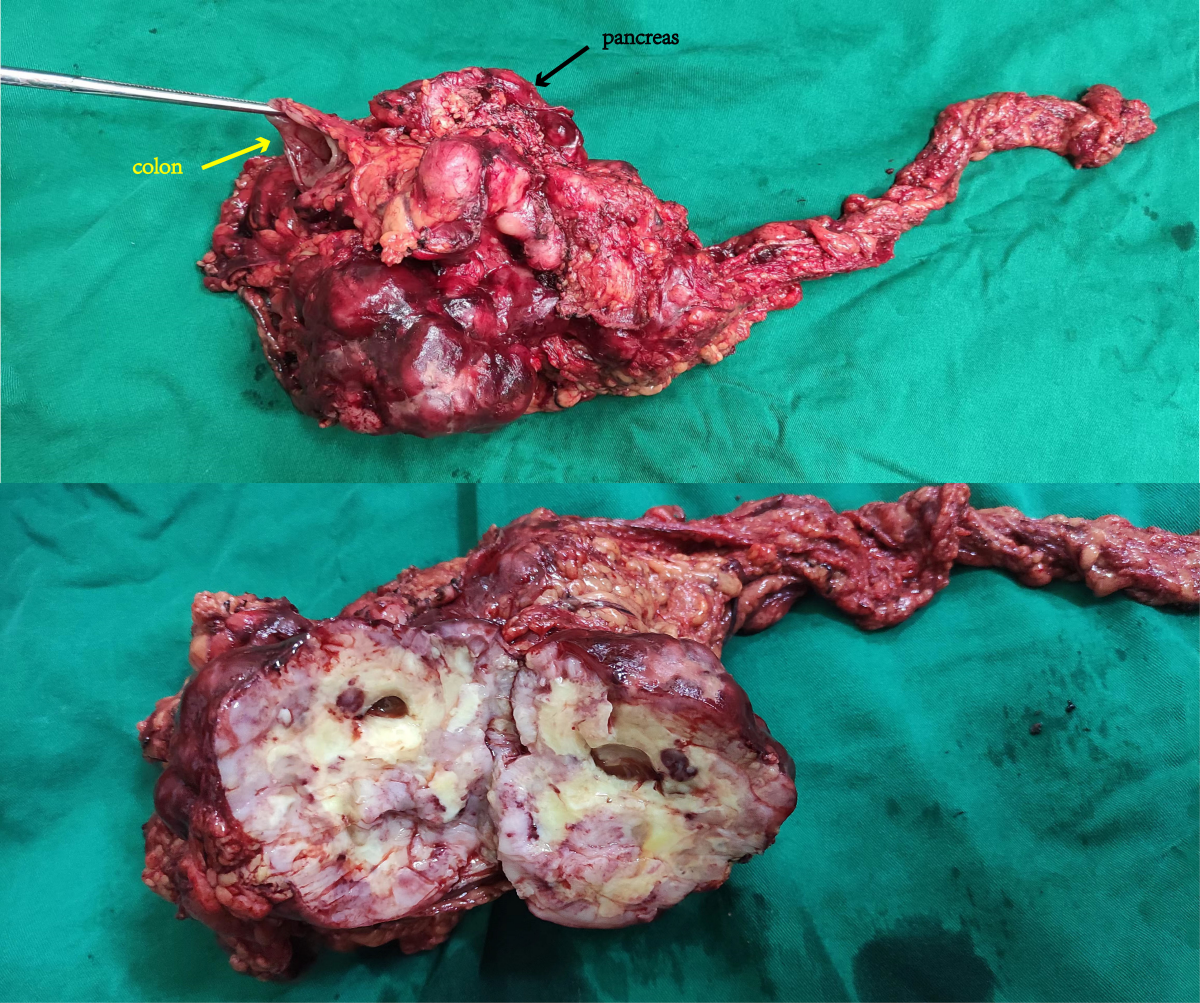
Grossly, the tumor is nodular, encapsulated, with gray-yellow cut surface and necrosis.

A 70-year-old man with a 10-year history of well-controlled hypertension was hospitalized due to an incidentally discovered abdominal mass. Physical examination revealed a large palpable mass with tenderness in the left upper quadrant. Abdominal and pelvic enhanced CT showed multiple soft-tissue density mass in the abdominal cavity, 14.1 cm×14 cm× 10.5 cm, lobulated, invading the stomach and pancreas. The tumor might come from the left upper abdomen and jejunum with multiple metastasis in the abdominal cavity. (**Figure 1**) Considering that the patient gradually developed incomplete intestinal obstruction during hospitalization, after a multi-disciplinary team (MDT) discussion, the patient accepted exploratory laparotomy, abdominal mass resection, left hemicolectomy and involved distal pancreatectomy, with splenic preservation. (**Figure 2**) Postoperative pathology showed that the tumor cells were short spindle and oval, and a large number of lymphocytes, plasma cells and eosinophils were infiltrated in the stroma. FISH staining showed isolated ALK signal in the nucleus. Based on the location, morphology, immunophenotype and molecular detection, the tumor was considered to be epithelioid inflammatory myofibroblastic sarcoma. The patient’s recovery was not uneventful, with a grade B pancreatic fistula and bilateral pleural effusion that improved after parenteral nutrition support, inhibition of pancreatic enzyme activity and drainage. In addition, the patient often experienced abdominal distension and occasional nausea postprandially, but no obvious signs of obstruction were seen on the postoperative plain CT scan of the abdomen and pelvis. The patient was discharged with an abdominal drainage tube while he was able to eat and move normally at 1 month after surgery.

One month after discharge, the abdominal drainage tube was removed in the outpatient department, but the patient still felt abdominal distension after eating. He received doxorubicin single-drug chemotherapy, but the regime could not be continued due to severe nausea and vomiting. He and his family were relatively satisfied with the treatment and accepted the follow-up advice. The patient was still alive without disease relapse at the 5-month follow-up after operation.

## Discussion

Epithelioid inflammatory myofibroblastic sarcoma (EIMS) is a malignant subtype of IMT, with high invasiveness and poor clinical prognosis (4, 7). The exact incidence of EIMS is still unknown, due to the rarity of EIMS, no large-scale, convincing clinical trial is available, with only ~55 cases being reported to date (4–6, 8–33). In this study, we describe an additional case of EIMS originating from the abdomen. The clinical characteristics of these 56 EIMSs are presented in **Table 1**.

**Table 1:** Clinical characteristics of 56 cases of epithelioid inflammatory myofibroblastic sarcoma.

case	age/sex	site	size(cm)	symptom	treatment	Recurrence	Metastasis	Follow up	Source
1	47/F	Mesentery of colon	6.5	Abdominal pain /distension	SE+RT	+	+	DOD(4M)	Wu.et al (22)
2	35/F	gastric	4	fatigue, anorexia	SE	–	–	NED(10M)	Xu.P et al (19)
3	14/M	retroperitoneal	18	abdominal pain	SE	+	+	DOD(3M)	Wan.et al (25)
4	45/M	abdominal cavity	20	Abdominal pain /distension	SE+Alk i	+	NA	DOD(NA)	Jiang. et al (11)
5	8/M	abdominal cavity	15.6	high fever	SE	NA	NA	DOD(8M)	Zhou. et al (27)
6	46/F	abdominal cavity	11	Abdominal pain /distension	SE+Alk i+AI	+	+	DOD(16M)	Zhang. et al (28)
7	65/M	Colon	9.3	Abdominal pain,hematochezia	SE+TCM	+	+	AWD(18M)	Bai. et al (29)
8	25/M	right lung	6.9	high fever,cough	ALK i+SE	–	+	AWD(12M)	Singh. et al (12)
9	21/M	left lung	10	fatigue,anorexia	SE+Alk i	–	+	DOD(4M)	Fu. et al (34)
10	43/F	uterus	7.8	abnormal uterine bleeding	SE	–	–	NED(60M)	Collins. et al (6)
11	22/M	ileum	6	abdominal pain,fatigue	SE+Alk i	+	+	AWD(14M)	Hosokawa. et al (15)
12	28/M	abdominal cavity	NA	abdominal distension,fatigue	ALK i	+	–	AWD(20M)	Xu X.J.et al (30)
13	26/M	pelvic cavity	17	abdominal pain /distension	CT+SE	NA	NA	NA	Du. et al (17)
14	0.4/F	abdominal cavity	11.4	abdominal mass	SE	–	–	NED(6M)	Batool. et al (31)
15	71/M	omentum	NA	NA	SE	+	–	AWD(6M)	Garg. et al (26)
16	19/F	lung	12.5	dyspnea, weight loss	SE+Alk i	+	–	AWD(12M)	Sarmiento. et al (20)
17	19/F	pelvic cavity	19	abdominal pain,nausea,vomiting	SE	+	–	DOD(3M)	Li. et al (23)
18	39/M	Mesentery of colon	15	dysuria	SE+CT	+	–	AWD(12M)	
19	34/M	liver	8	abdominal distension	SE	+	–	DOD(5.5M)	Lee. et al (10)
20	62/M	abdominal cavity	25	NA	SE+CT	+	–	DOD(2M)	
21	76/F	abdominal cavity	9	NA	SE	+	–	DOD(4M)	
22	30/M	abdominal cavity	10	NA	SE	+	–	DOD(8M)	
23	16/F	lung	8	NA	SE+CT+RT+ALK i	+	–	AWD(48M)	
24	42/M	abdominal cavity	NA	NA	NA	+	–	AWD(40M)	
25	26/M	abdominal cavity	NA	NA	NA	+	–	AWD(16M)	
26	39/F	abdominal cavity	NA	NA	NA	+	+	AWD(10M)	
27	72/F	temporal lobe	4.7	NA	SE+ALK i	+	+	NA	
28	44/M	abdominal cavity	NA	abdominal pain	SE+Alk i+CT	+	–	AWD(28M)	Butrynski. et al (18)
29	57/M	thoracic cavity	NA	dyspnea	Alki	NA	NA	NA	Kozu Y. et al (5)
30	15/F	ovary	NA	Abdominal pain	SE+Alk i+CT	+	–	AWD(24M)	Fang. et al (8)
31	7/M	abdominal cavity	NA	Abdominal pain	SE+CT	+	–	AWD(5M)	Ma. et al (24)
32	0.7/M	abdominal cavity	11	abdominal distention	SE+CT	+	–	AWD(5M)	
33	35/F	abdominal cavity	11	Abdominal mass, weight loss	SE	–	–	NED(6M)	Huang. et al (21)
34	2/M	Retroperitoneal	11	abdominal mass	SE	–	–	NED(36M)	Patel. et al (32)
35	42/F	abdominal cavity	19	Abdominal pain/distension	SE+Alk i	+	–	26M(AWD)	Wang. et al (13)
36	27/F	groin	1.5	node	SE	–	–	NED(12M)	Gadeyne.et al (35)
37	55/F	abdominal cavity	20	abdominal discomfort	SE+CT	–	–	NED(14M)	Rafee. et al (14)
38	53/F	pericardium	NA	dyspnea	SE+CT	+	–	DOD (2M)	Azad. et al (9)
39	NA	sigmoid(colon)	11.9	Leukocytosis	SE+ALKi	NA	NA	NA	Liu. et al (33)
40	14.7/NA	pelvic cavity	NA	NA	SE+Alk i	+	–	AWD(72M)	Trahair T, et al (16)
41	11.3/NA	abdominal cavity	NA	NA	SE+Alk i	–	–	NED(48M)	
42	9.1/NA	abdominal cavity	NA	NA	ALK i	+	–	DOD(11M)	
43	1.4/NA	abdominal cavity	NA	NA	Alk i+SE	–	–	NED(9M)	
44	22/M	Mesentery of colon	10.4	Abdominal pain, fever	SE+Alk i	–	–	NED(16M)	
45	59/M	mesentery	15	NA	SE+CT	+	–	DOD(12M)	Marino-Enriquez A
46	41/M	omentum	26	NA	SE+CT+Alk i	+	+	NED(40M)	.et al (4)
47	6/M	omentum	10.5	NA	SE+CT	+	–	AWD(13M)	
48	28/M	mesentery	NA	NA	NA	NA	NA	NA	
49	63/M	mesentery	25.5	NA	SE+CT	+	–	DOD(3M)	
50	42/M	abdominal cavity	NA	NA	SE+CT	+	–	AWD(13M)	
51	0.7/M	peritoneum	10	NA	SE+CT+RT	+	–	DOD(36M)	
52	40/M	peritoneum	8	NA	SE+CT+RT	+	+	DOD(28M)	
53	31/F	mesentery	17.5	NA	SE+CT	+	–	DOD(11M)	
54	6/M	omentum,mesentery	14	NA	SE	NA	NA	NA	
55	39/M	mesentery	15	NA	SE	NA	NA	NA	
56	70/M	abdominal cavity	14	NA	SE+CT	–	–	NED(5M)	current case

ALKi, ALK inhibitor; AWD, alive with disease; CT, chemotherapy; DOD, dead of disease; NA, not available; NED, no evidence of disease; RT, radiation therapy; SE, surgical excision; AI.

–, No; +, Yes.

The onset age of EIMSs ranges from 4 months to 76 years (with mean age 31.6 years), and tends to occur in men and within the abdominal cavity. The reported sites of involvement include the liver (36), rectum, and transverse colon (37). The lung is also a common site of involvement (34). In addition, rare sites such as ovary (8), inguinal subcutaneous tissue (38), central nervous system (39) and pericardium (9) have also been reported. In a review of the previous literature, symptoms of EIMS have been associated with tumor location without significant specificity. Instead of abdominal pain and palpable masses, systemic fatigue and weight loss are the main clinical manifestations in some patients (34). In addition, dyspnea, cough, bleeding, repeated fever, and leukemia-like reaction have been reported as the first symptoms.

The diagnosis of EIMS is challenging because of its rarity, atypical clinical symptoms, and no characteristic findings on imaging examination. Additionally, the low positive rate of biopsy, and the atypical morphological features add to the difficulty of diagnosis. Previous studies have also proposed diagnostic criteria including: (1) round-to-epithelioid tumor cells; (2) plentiful myxoid stroma with inflammatory infiltration; (3) positive ALK immunohistochemical test (5). Nevertheless, the diagnosis of EIMS based on histology or ALK expression alone may be precarious as not all IMTs of epithelioid/round cell morphology carry the genetic changes of EIMS (2), and some mesenchymal tumors such as rhabdomyosarcoma, lipoma, leiomyosarcoma, malignant peripheral nerve sheath tumor, Ewing sarcoma/peripheral primitive neuroectodermal tumor, etc. may also have positive ALK cytoplasmic staining (40, 41). Consequently, it is recommended that further detection of ALK rearrangements by FISH, RT-PCR or next-generation sequencing(NGS) techniques be performed to confirm the diagnosis of EIMS in cases with atypical morphology or unusual immunoprofile.

Strong desmin positivity and perinuclear or cytoplasmic ALK positivity were confirmed in all described EIMS cases (39). Furthermore, ALK can be fused with multiple genes to form different chromosomal rearrangements in IMT, including TPM3 and TPM4, CLTC, RAN-BP2, CARS, ATIC, and SEC31L1. The most common form of EIMS is RANBP2-ALK fusion, which is characterized by nuclear membrane staining of ALK protein on immunohistochemistry. RRBP1-ALK (10) fusion and EML4-ALK (11) fusion have also been reported. According to the difference in fusion gene and the location of fusion protein, the displayed immunohistochemical positive pattern is different. Since RANBP2 was a macromolecular protein located in the nuclear pore, the positive position of ALK protein fusion mode by immunohistochemistry was located in the nuclear membrane. RRBP1 is a ribosomal binding protein, and the positive position by RRBP1-ALK fusion immunostaining is cytoplasmic and perinuclear. Both are associated with more aggressive biological behavior and a worse prognosis (10, 12, 37). In addition, VCL-ALK gene fusion has also been reported in the central nervous system EIMS (39).

The EMIS should be differentially diagnosed from other disease as follows: (1) Anaplastic large cell tumor (ALCL), especially the sarcomatoid variantis, the most difficult to differentiate from EIMS because they have similar histomorphology and are positive for ALK, CD30, and SMA (42). However, RANBP2-ALK fusion and typical ALK nuclear membrane expression pattern were not observed in ALCL, and most of them were expressed in the cytomembrane and Golgi apparatus. In addition, strong desmin positivity was absent in ALCL.(2) High-grade leiomyosarcoma lack of typical expression pattern of ALK in EIMS, but is generally accompanied with typical histological features in focal region, that is spindle cells arranged in bundles with red-stained cytoplasm and “cigar-shaped” nuclei (3) A solid variant of alveolar rhabdomyosarcoma, often ALK-positive (40), could usually be differentiated by histological examination. It is generally homogeneous cytologically, with inadequate cytoplasm, a lack of myxoid stroma, and prominent neutrophils. Nuclear immunopositivity for myostatin (MYFF 4) contributed to the diagnosis of rhabdomyosarcoma.(4) Dedifferentiated liposarcoma, especially those showing an “inflammatory MFH” pattern (43). Dedifferentiated liposarcoma can also mimic conventional IMT (44, 45). In such cases, overexpression of MDM2 can be misleading, as a high proportion of IMTs show nuclear staining of MDM2 (3, 46), however, the nuclear membrane staining for ALK is absent in liposarcoma.(5) Gastrointestinal stromal tumor (GIST), although epithelioid GISTs with inflammatory myxoid backgrounds are rare, GISTs should be ruled out first for mesenchymal tumors in the abdominal cavity, which can be identified by immunohistochemistry CD117, CD34, and Dog-1. Furthermore, GIST are associated with C-kit and PDGF-α mutations.

There is no consensus on the optimal treatment for EIMS, owing to its rarity and high malignancy, and surgical resection is still considered the primary treatment. However, recurrence is common after resection (13, 36). Some previous reports indicated that postoperative adjuvant chemotherapy or radiotherapy has no distinct effect in the control of aggressive progression of EIMS (4, 14–16, 37). Postoperative immunotherapies have not yet been clearly identified. Programmed cell death ligand 1 (PD-L1) is found to be diffusely positive in some cases (17). The PD-1/PD-L1 axis has also been reported to play an important role in the immune antitumor response (47, 48). Therefore, a new immunomodulatory therapy targeting the PD-1/PD-L1 pathway might be developed.

It is reported that crizotinib has strong and durable activity against ALK-positive IMT (49, 50). Since ALK gene rearrangement has been reported in all cases, ALK inhibitors such as crizotinib and ceritinib may display unexpected therapeutic prospects for EIMS. Butrynski et al. described for the first time the therapeutic effects of crizotinib in patients with RANBP2-ALK fusion EIMS (18). Trahair, T et al. suggested that crizotinib is tolerable and effective in patients with life-threatening complications (16). Subsequent reports have also shown a relatively satisfactory effect of ALK inhibitors in patients with EIMS (10, 15, 37). At the same time, ALK negative patients didn’t respond (10, 19). However, long-term reactions or responses are seldom seen and most patients with EIMS experience disease relapse, progression, or metastasis within three to six months after receiving crizotinib monotherapy (5, 20, 34). Furthermore, studies by Trahair, T et al. have shown that patients with RANBP2-ALK rearrangement EIMS still relapse despite a complete response to crizotinib initially (16). Alectinib, a highly selective ALK inhibitor, has been shown to have a high response rate and a long progression-free survival in patients with ALK-positive lung cancer in Phase I/II studies, without severe toxicity, even in patients who have failed treatment with crizotinib. In a phase III study, compared with crizotinib, alectinib showed superior efficacy and lower toxicity in primary treatment of ALK-positive NSCLC, which may suggest a more potent effect of aletinib-bick-zotinib in patients with EIMS.

CD30 positivity is another characteristic pathological feature of EIMS (4, 16, 21, 22, 35), suggesting that brentukimab vedotin (BV), a CD30-targeting antibody-drug conjugate, could be a potential therapeutic option for EIMS. Additionally, Fordham, A.M. et al. validated the potential of CD30 as a therapeutic target in EIMS, and the combination therapy targeting CD30 and ALK at the time of initial treatment was more effective than monotherapy or combination therapy at the time of relapse, which also suggests the potential of combination therapy in preventing recurrence or treating ALKi-resistant EIMS disease (51).

Regarding the biological behavior of EIMS, of the 48 patients with follow-up information, 18 (37.5%) died of the disease (15 within 1 year of diagnosis and 3 within 3 years of diagnosis), 19 (39.5%) were alive with the disease, and the remaining 11 (23%) were well without evidence of disease. The median overall survival was 12 months (mean 17.4 months). Furthermore, Only 8(16.7%) patients were followed up without recurrence, disease progression or metastasis. To date, clinicopathological factors associated with the prognosis of EIMS are unknown. Multiple studies have suggested that the aggressive course of the disease may be related to intraperitoneal origin, large tumor size, epithelioid morphology and RANBP2-ALK (2–4, 18, 23, 24).

Despite the favorable efficacy of ALK inhibitors such as crizotinib in the treatment of EIMS, there are still many unresolved issues that need to be further explored. For instance, it is currently unclear whether crizotinib should be used as first-line therapy or whether surgery should be performed before crizotinib treatment to minimize tumor size. In our opinion, ALK inhibitor therapy may be given priority to patients who need combined multiple organ resection after preoperative evaluation, because such patients usually have a poor prognosis and have many serious postoperative complications (25, 34). One of the current issues to be addressed is how to prevent or delay the development of resistance to ALK inhibitors. Further studies are needed in patients with EIMS. Moreover, further drug development is necessary to increase the life expectancy of patients and alleviate the disease.

## Conclusion

In conclusion, we present a case of primary abdominal EIMS, a highly aggressive variant of IMT characterized by epithelioid cell morphology, often with inflammatory infiltration of neutrophils and prominent perinuclear or nuclear membrane ALK staining. It has a significant male advantage and appears in the intra-abdominal region. The diagnosis of these tumors can be difficult due to epithelioid morphological abnormalities, especially when the tumor site is atypical. In addition, we show how this can be distinguished from its histological mimicry. The detection of ALK rearrangement by FISH, RT-PCR or NGS will contribute to further clarify the diagnosis of EIMS. Furthermore, the idefication of EIMS has important implications for clinical management, as ALK inhibitors are critical and effective agents for EIMS treatment.

## Data availability statement

The original contributions presented in the study are included in the article/supplementary material. Further inquiries can be directed to the corresponding authors.

## Ethics statement

Written informed consent was obtained from the individual(s) for the publication of any potentially identifiable images or data included in this article.

## Author contributions

WD drafted the manuscript. YG, TL, HZ, SF, YW collected data. ZL and XW designed the study and revised the manuscript. All authors contributed to the article and approved the submitted version.
